# Increased DNA typing success for feces and feathers of capercaillie (*Tetrao urogallus*) and black grouse (*Tetrao tetrix*)

**DOI:** 10.1002/ece3.3951

**Published:** 2018-03-23

**Authors:** Stephanie Vallant, Harald Niederstätter, Burkhard Berger, Reinhard Lentner, Walther Parson

**Affiliations:** ^1^ Institute of Zoology University of Innsbruck Innsbruck Austria; ^2^ Institute of Legal Medicine Medical University of Innsbruck Innsbruck Austria; ^3^ Department of Environmental Protection Provincial Government of Tyrol Innsbruck Austria; ^4^ Forensic Science Program Pennsylvania State University University Park PA USA

**Keywords:** microsatellite genotyping, noninvasive sampling, short tandem repeat, *Tetrao tetrix*, *Tetrao urogallus*

## Abstract

Noninvasive sampling, for example, of droppings or feathers, is a promising approach for molecular genetic studies on endangered and elusive animal species. Yet, such specimens are known for containing only minute amounts of DNA, resulting in lower typing success rates relative to analyses on fresh tissues such as muscle or blood. Furthermore, artefactual signals as well as contamination are more likely to occur when DNA is limited. To increase the reliability of DNA typing from noninvasive samples, optimized DNA extraction and polymerase chain reaction protocols were developed, taking advantage of developments in the forensic field aiming at successful molecular genetic analysis of DNA templates being low in quality and quantity. In the framework of an extensive monitoring project on population dynamics of capercaillie and black grouse in the Tyrolean Alps, feces samples and molted feathers from both species were collected. On a subset comprising about 200 specimens of either species, eight polymorphic short tandem repeat (STR) markers were analyzed to test these improved protocols. Besides optimizing DNA yields, both lowered sample consumption and reduced hands‐on time were achieved, and the rates of informative profiles amounted to 90.7% for capercaillie and 92.4% for black grouse. Similarly, high success rates had not been achieved in earlier studies and demonstrate the benefit of the improved methodology, which should be easily adaptable for use on animal species other than those studied here. The STR genotypes were not only powerful enough to discriminate among unrelated birds but also appeared fit for telling apart closely related animals, as indicated by Pi and Pi_sib_ values. The software package allelematch aided analysis of genotypes featuring possible dropout and drop‐in effects. Finally, a comparison between molecular genetic and morphology‐based species‐of‐origin determination revealed a high degree of concordance.

## 1 INTRODUCTION

Conservation and population genetic studies are indispensable sources of information in decision‐making processes regarding environmental management and the protection of endangered species. For suitable management and protection plans, reliable data on population sizes, trends, and conservation status are required.

**Figure 1:**
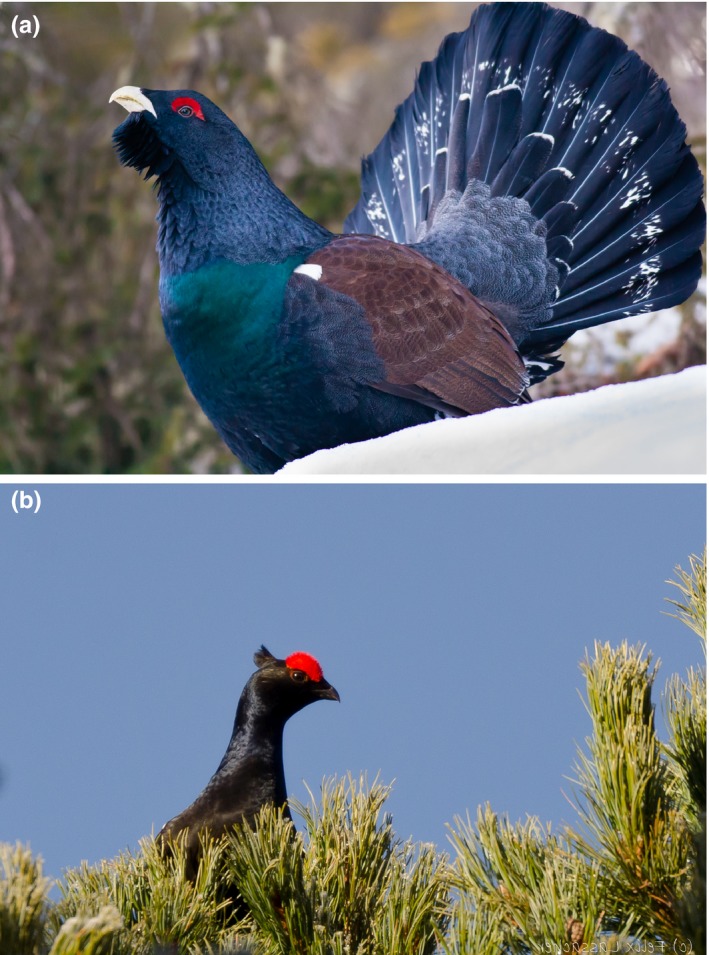
(a) Displaying male capercaillie around the lek site (*Tetrao urogallus,* photograph SV). (b) Male black grouse (*Tetrao tetrix*) displaying and calling in a tree top (photograph Felix Lassacher)

To date, in Tyrol conservation and population studies on capercaillie (*Tetrao urogallus* Linnaeus) and black grouse (*Tetrao tetrix* L.)—two endangered bird species (Dvorak & Ranner, 2014; IUCN 2014; Landmann & Lentner, 2001)—are mainly based on counts of males at known leks (Reimoser & Wildauer, 2006). Leks are sites where these animals aggregate during the mating season in spring and males display to attract females (Figure 1; Höglund & Alatalo, 1995). However, count‐based population size estimates have been considered inappropriate for assessment of long‐term population trends (Cayford & Walker, 1991; Jacob, Debrunner, Gugerli, Schmid, & Bollmann, 2010). More recently, molecular genetic approaches have been developed for a wide range of biological systems and were rated superior to traditional counting by providing less biased data (Regnaut, 2004). DNA analysis allows monitoring of individuals through genetic fingerprinting (Caizergues, Dubois, Loiseau, Mondor, & Rasplus, 2001; Jacob et al., 2010; Piertney & Höglund, 2001; Segelbacher, Paxton, Steinbrück, Trontelj, & Storch, 2000).

A downside of routine genotyping approaches is that they typically require a direct sampling approach involving capture of the animals and invasive collection of DNA sources such as blood or muscle. This may cause unacceptably high levels of disturbance (Regnaut, 2004). On the contrary, collecting noninvasive sampling material (e.g., feces, feathers, or hairs) in an “indirect sampling approach” largely avoids unwanted interactions with the studied animals (Segelbacher, 2013; Taberlet, Waits, & Luikart, 1999). However, establishing reliable genotypes can be a nontrivial task, as DNA extracted from noninvasive samples may be (very) low both in quantity and in quality. For instance, fecal samples may not only contain just minute amounts of template DNA but also constitute a source of polymerase chain reaction (PCR) inhibitors.

Allelic dropout phenomena and/or false alleles are more likely to occur with such samples, and contamination is more of a concern with low‐copy‐number templates (Taberlet et al., 1999). Thus, compared to directly drawn samples (e.g., blood), noninvasively collected specimens can be considered a more difficult matrix for DNA extraction. As a consequence, genotyping success rates tend to be lowered and, to avoid generation of phantom individuals by declaring flawed profiles unique, error needs to be accounted for. Extraction from challenging specimens should, therefore, yield the highest possible DNA quality and quantity to facilitate reliable genotyping in downstream analyses. By taking advantage of research in forensic genetics aiming at optimized DNA extraction and multiplexed DNA analysis techniques, extraction methods for feces and feathers were optimized and applied to a molecular genetic study on free‐ranging capercaillie and black grouse in Tyrol.

## 2 MATERIALS AND METHODS

### 2.1 Study sites and sample collection

**Figure 2:**
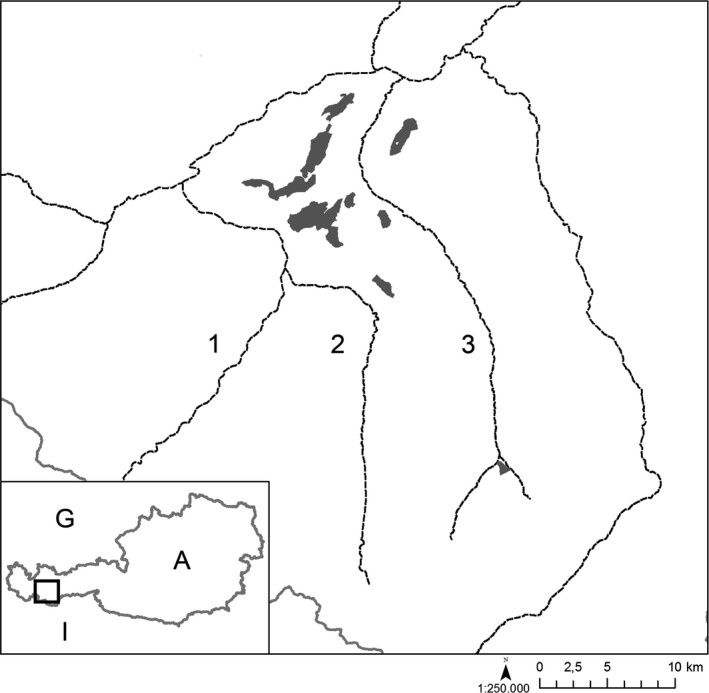
Study sites (shaded) in the Inner Alps in the western part of Tyrol. Rivers are shown as dashed lines. 1: Inn Valley, 2: Kauner V., 3: Pitz V.; A: Austria, G: Germany, I: Italy

The study area was located in the Inner Alps of western Tyrol, Austria (Figure 2). It was mainly situated in the subalpine and montane zones and ranged from 1,350 to 2,300 m above sea level. The arboreous area was heavily dominated by *Picea abies,* and the forest boundary consisted mainly of *Larix decidua* and *Pinus cembra* (Pitschmann, Reisigl, Schiechtl, & Stern, 1980).

Species‐specific habitat models based on Graf and Bollmann (2008) and Masoner (2012) were used to identify priority habitats for capercaillie and black grouse and to define areas for intense field collection. According to background information provided by local hunters, all such chosen areas‐of‐interest contained known lek sites. Systematic sampling of capercaillie and black grouse feces and feathers covered the entire area of interest, and lek site information was not forwarded to the field surveyors.

Fieldwork took place between March and May 2012, and study sites were visited twice within a period of 10–14 days. During this sampling period, both black grouse and capercaillie aggregated around lek sites for mating, which aided representative sampling of the local populations (Höglund & Alatalo, 1995).

Per day and surveyor, an area of 56–139 ha was sampled along transects spanning the entire width of the study site and being 100 m apart from each other. However, in reaction to local conditions in topography, this uniform pattern was re‐adjusted if necessary. Furthermore, field surveyors were allowed to accommodate their tracks for covering suitable habitat structures. A short description of the recovery site along with its GPS coordinates was recorded for every specimen.

Based on our experience, the cooling effect of snow appears to decrease biodegradation and thus increases chances of successful DNA typing. Therefore, sample collection was restricted to feathers appearing intact and to feces on snow or displaying a visibly moist surface. All specimens were stored at −20°C until further use.

Initial morphological species assignments were performed by trained field surveyors on‐site.

### 2.2 Voucher specimens

Biological reference material for both bird species was provided by the Alpenzoo Innsbruck (Innsbruck, Austria). After collection from the wing vein by a staff veterinarian, blood of capercaillie (one individual of each sex) and black grouse (one male, two females) was air‐dried on Whatman FTA blood stain collection cards (GE Healthcare, Chalfont St Giles, UK) and stored at ambient temperature in the dark. Additionally, fresh feathers and feces were collected from all reference birds and stored at −20°C.

### 2.3 Ethics statement

With prior notice, consultation, and agreement by the person entitled to the hunting right, the permission to enter the hunting grounds was granted for the purpose of conducting this study. As the aim of our study was not to find the birds themselves but to provide noninvasive sampling material, field surveyors only searched for droppings and feathers. Therefore, the disturbance of the investigated birds was kept to a minimum, and no specific permission to work with living animals was needed. Reference material of voucher animals was collected by a veterinarian in the “Alpenzoo” (Innsbruck, Austria) during a routine veterinary examination required by the law.

### 2.4 DNA extraction

DNA extractions followed best practice lab routines and were performed in a dedicated pre‐PCR area. Extraction blanks were run along the specimen of interest.

DNA from blood samples was extracted using the DNeasy Blood & Tissue kit (Qiagen, Hilden, Germany) following the manufacturer`s instructions. For feather samples, we used the EZ1 DNA Investigator kit (Qiagen). Therefore, 1 cm of the basal part of the feather was cut into small pieces as described by Segelbacher (2002). Lysis of large feathers (i.e., remiges and rectrices) was performed overnight at 56°C under constant shaking (550 rpm) in 1,000 μl TN_ca_ buffer comprising 10 mmol/L Tris‐HCl pH 8.0, 100 mmol/L NaCl, 1 mmol/L CaCl_2_, 2% SDS (w/v), 40 μl proteinase K (>600 mAU/ml, Qiagen) and 40 μl dithiothreitol (1 mol/L, Qiagen) as described by Hellmann, Rohleder, Schmitter, and Wittig (2001). For smaller feathers, the lysis cocktail was halved in volume. Further steps were conducted according to manufacturer's instructions (EZ1 DNA Investigator kit large volume protocol).

DNA extraction from feces was performed with the DNA Stool Mini kit (Qiagen) according to the vendor's protocol with modifications as specified below. Using sterile cotton sticks (Applimed, Châtel‐Saint‐Denis, CH), dry swabs were taken from the surface of the samples. Regions with visible traces of uric acid were avoided (Segelbacher & Steinbrück, 2001). Cotton swabs were cut, and digestion was conducted overnight at 56°C in a shaker (550 rpm; Segelbacher G., personal communication, February 12, 2013). Subsequently, 4 μl carrier RNA (1 μg/μl, Qiagen) was added, and the DNA was bound to the provided silica membrane (QIAamp spin column, Qiagen). After two washing steps with the provided buffer, the DNA was sequentially recovered in 2 × 75 μl buffer AE (Jacob et al., 2010) and stored at −20°C.

### 2.5 STR markers

For genetic individualization, the six short tandem repeat (STR) loci TuT1, TuT2, TuT3, TuT4, TuD1, and TuD6 were used (Segelbacher et al., 2000). Primers amplifying short fragments (Jacob et al., 2010) were utilized where possible (Table 1). Additionally, the STR loci BG15 and BG18 (Piertney & Höglund, 2001) were included in this study. For this, the amplification primers’ 5’ ends had to be modified slightly (Table 1). The resulting marker set was typed in three multiplex PCR assays (MP‐1, MP‐2, and MP‐3) using fluorescently labeled primers (Table 1).

**Table 1:** Primer sequences, fluorescent label, and repeat motif information for PCR multiplexes

Multiplex PCR	Locus	Forward primer	Reverse primer	Motif	Ref
MP‐1	sTuT2	FAM‐TCTCCAAACTAGATATGGAAACCAG	CAAAGCTGTGTTTCATTAGTTGAAG	GATA	Jacob et al. (2010)
	mTuT1^a^	GGTCTACATTTGGCTCTGACC	VIC‐GCACAGGAACAGCAATAGATGG	CTAT	Jacob et al. (2010), Segelbacher et al. (2000)
	BG18	NED‐CGCCATAACTTAACTTGCACTTTC	CTTCCTGATACAAAGATGCCTACAA	CTAT	Piertney and Höglund (2001)
	sTuT3	GCCTCAACTAATCACCCCTTTATC	PET‐GAGGGATTTATGCATGCTGCTAG	TATC	Jacob et al. (2010)
MP‐2	BG15	FAM‐GAATAAATATGTTTGCTAGGGCTTAC	GATCTTACATTTTTCATTGTGGACTTC	CTAT	Piertney and Höglund (2001)
	sTuD6	VIC‐AGCCTTTTACTGCACTACTTGC	GGTGTGTGGGAAATGAGGAC	CA	Jacob et al. (2010)
MP‐3	sTuD1	FAM‐ATTTGCCAGGAAACTTGCTC	CCTTTGCCTCCTTATGAAATCC	CA	Jacob et al. (2010)
	sTuT4	NED‐TGGGAGCATCTCCCAGAGTC	ACAAACAAGGCAGCAGCATG	TATC	Jacob et al. (2010)

a Locus was denoted with **m** for modified, forward primer: Segelbacher et al. (2000), reverse primer: Jacob et al. (2010).

### 2.6 PCR amplification and electrophoretic product sizing

PCR amplification was carried out in 96‐well polypropylene PCR plates. The 10 μl reactions contained 5 μl 2× multiplex PCR kit master mix (Qiagen), 1 μl nonacetylated bovine serum albumin (2.5 μg/μl; Sigma‐Aldrich, St. Louis, MO), 4 mmol/L MgCl_2_, 160 nmol/L of each primer, and 1 μl of template DNA.

PCR amplifications were performed on a GeneAmp PCR System 9700 thermal cycler (LT, Life Technologies, Carlsbad, CA). Thermal cycling comprised of initial denaturation for 15 min at 94°C, followed by 37 cycles of denaturation for 94°C at 30 s, primer annealing at 56°C (MP‐1 and MP‐3) or 58°C (MP‐2) for 120 s, and primer elongation at 72°C for 30 s. The final elongation step at 72°C was extended by 45 min. Negative (extraction blanks, no template controls) and positive controls (diluted DNA of voucher specimens) were amplified and analyzed along the samples of interest.

Electrophoretic sizing of amplicons was conducted on an ABI 3100 Automated Sequencer using POP‐6 (both LT). An aliquot of the PCR product (1 μl) was diluted in 20 μl Hi‐Di formamide (LT) including 0.3 μl GeneScan 500 LIZ internal size standard (LT) and denatured at 95°C for 3 min. Following electrophoretic separation, amplicon sizes were estimated using the GeneMarker HID 1.7 software (SoftGenetics, State College, PA). Allele calling was based on the apparent amplicon length (bp) rounded to the next integer.

Profiles featuring data for all eight loci are dubbed “full” hereafter, whereas partial profiles comprising information for 5–7 loci only were considered “informative.”

### 2.7 Molecular genetic species identification

Molecular genetic allocation to capercaillie or black grouse relied on species indicative allele size ranges observed for markers BG15, BG18, and sTuT2 (Jacob et al., 2010; G. Segelbacher, personal communication, February 12, 2013). Furthermore, using these loci, two other local grouse species, hazel grouse (*Tetrastes bonasia* L.) and rock ptarmigan (*Lagopus muta* Montin), can be identified, too (G. Segelbacher, personal communication, February 12, 2013).

### 2.8 Individualization of samples

The allelematch package (Galpern, Manseau, Hettinga, Smith, & Wilson, 2012) for R (version 3.1, R Development Core Team 2014) was used for identifying unique multilocus genotypes. This software enables the assignment of genotypes to individuals by also considering the possibility of missing or compromised data (Galpern et al., 2012). The R script utilized is to be found in the supporting information accompanying this study (Appendix S1).

### 2.9 Statistical analyses

Allele frequencies, probability of identity (Pi), and probability of identity for siblings (Pi_sib_) were estimated with GENECAP [version 1.4; (Wilberg & Dreher, 2004)]. Pi defines the likelihood of two randomly chosen, unrelated individuals sharing the same genotype, and Pi_sib_ describes the probability of two identical genotypes in siblings (Waits, Luikart, & Taberlet, 2001). ARLEQUIN [version 3.5.1.2; (Excoffier, Laval, & Schneider, 2005)] was used to calculate expected and observed heterozygosity values and to test for deviation from Hardy–Weinberg equilibrium (HWE) for all loci. Statistical inference was based on a critical type‐I error probability of .05 for rejecting the null hypothesis. When performing multiple comparisons, Bonferroni correction was employed to adjust this critical *p*‐value. Microchecker (version 2.2.3; Van Oosterhout, Hutchinson, Wills, & Shipley, 2004) was utilized to check the genotypes for potential null alleles. To test the reliability of genotyping, twelve samples were chosen for both species. These subsamples comprised to equal parts randomly selected specimens featuring full (data for all 8 loci) and partial, yet informative, profiles (data for 5–7 loci) in the initial round of genotyping. Multiplex PCR analysis was replicated independently six times for these samples. Error rates were estimated with GIMLET [version 1.3.3; (Valière, 2002)]. Input files used in statistical analyses are to be found in the Data Files S1 and S2.

## 3 RESULTS

### 3.1 STR genotyping of voucher animals

For capercaillie and black grouse, voucher specimens of both sexes were genotyped using three different types of biological reference material (feces, feathers, blood). As expected, these sample types yielded (1) full STR profiles (i.e., successful amplification of all eight targeted microsatellite loci), and (2) perfectly matching intra‐individual genotyping results. Furthermore, our results for capercaillie and black grouse confirmed species indicative allele sizes at loci BG15, BG18, and sTuT2 (Tables 2 and S1).

**Table 2:** Allelic range and detected alleles at eight STR loci and results of HWE testing

Locus	Capercaillie (*n* = 226)							Black grouse (*n* = 210)						
	A	AR	Pi	Pi_sib_	H_O_	H_E_	*p*	A	AR	Pi	Pi_sib_	H_O_	H_E_	*p*
sTuT2	8	140–168	0.18	0.49	0.63	0.62	.4947	3	119–131	0.58	0.77	0.25	0.25	.8400
mTuT1	5	182–198	0.12	0.42	0.41	0.73	**<.0001**	9	166–198	0.06	0.37	0.39	0.81	**<.0001**
BG18	6	197–217	0.16	0.45	0.71	0.68	.9390	8	149–177	0.09	0.39	0.69	0.78	.6197
sTuT3	5	88–108	0.25	0.51	0.70	0.60	.2209	7	100–124	0.11	0.40	0.70	0.73	.0960
BG15	6	136–156	0.14	0.44	0.71	0.71	.2320	9	172–208	0.08	0.38	0.64	0.78	**.0010**
sTuD6	13	144–186	0.04	0.34	0.90	0.86	.0833	8	146–160	0.12	0.42	0.62	0.72	**.0025**
sTuD1	7	150–164	0.10	0.41	0.69	0.75	.0321	6	154–164	0.27	0.56	0.41	0.52	**.0057**
sTuT4	5	126–142	0.18	0.48	0.62	0.64	.1263	9	118–146	0.05	0.35	0.64	0.83	**.0002**

Species indicative apparent allele size ranges for hazel grouse were 158–190 bp (sTuT2), 132–240 bp (BG15), and 138–160 bp (BG18). Those of rock ptarmigan were 124–160 bp (sTuT2), 148–164 bp (BG15), and 148–184 bp (BG18). A, number of different alleles; AR, allelic range (given as apparent length in base pairs; Pi, probability of identity; Pi_sib_, probability of identity for siblings; H_o_, observed heterozygosity; H_E_, expected heterozygosity for each locus; values deviating statistically significantly from HWE after Bonferroni correction are indicated in bold. Note: 5’‐terminal trimming of amplification primers resulted in shorter amplicons for markers BG15 (−10 bp) and BG18 (−6 bp) compared to Jacob et al. (2010).

### 3.2 STR genotyping of field samples

Based on the initial species assignment relying on morphological features of feces and feathers, a subset of 471 of the total 877 collected samples was selected for DNA analysis. Conditions of selection were as follows: (1) the number of samples was proportional to the area of the study site, and (2) in a radius of 30 m, only one of the collected samples was considered to reduce the risk of excessive resampling the same individuals. Typically, feces samples were preferred over feathers due to higher chance of genotyping success. The resulting subsample comprised 227 (217 feces and 10 feathers) and 235 (184 feces and 51 feathers) samples initially assigned as capercaillie and black grouse, respectively, and nine (eight feces and one feather) samples with inconclusive morphological species assignment.

This subset of 471 field‐collected specimens was STR genotyped. Mean percentage of positive PCR over all loci was 88.03 ± 1.22 (*SD*). The entire set of loci proved to be polymorphic and detailed results can be found in Tables [Link], [Link], [Link], [Link]. Only seven samples (1.5%, three feces and four feathers) did not produce detectable levels of any specific PCR product. Further, due to failed amplification of species indicative loci, another 15 samples (3.2%, eight feces and seven feathers) could not be assigned to a species and were not further considered for analysis. Hence, 449 samples (95.3%) remained in the study.

#### 3.2.1 Molecular genetic species identification

Based on the species indicative BG15, BG18, and sTuT2 allele sizes (Tables 2, S1, S4, and S5) obtained for these 449 samples, we attributed 226 samples (50.3%) to capercaillie and 210 (46.8%) to black grouse. Two other local grouse species were identified in the analysis. One (0.2%) sample originated from hazel grouse and 12 (2.7%) samples from rock ptarmigan. Further analyses described here were restricted to capercaillie and black grouse genotypes, all other samples from hazel grouse and rock ptarmigan were excluded as their habitats were not systematically examined during field studies.

#### 3.2.2 Comparison of genetic and morphological species identification

**Figure 3:**
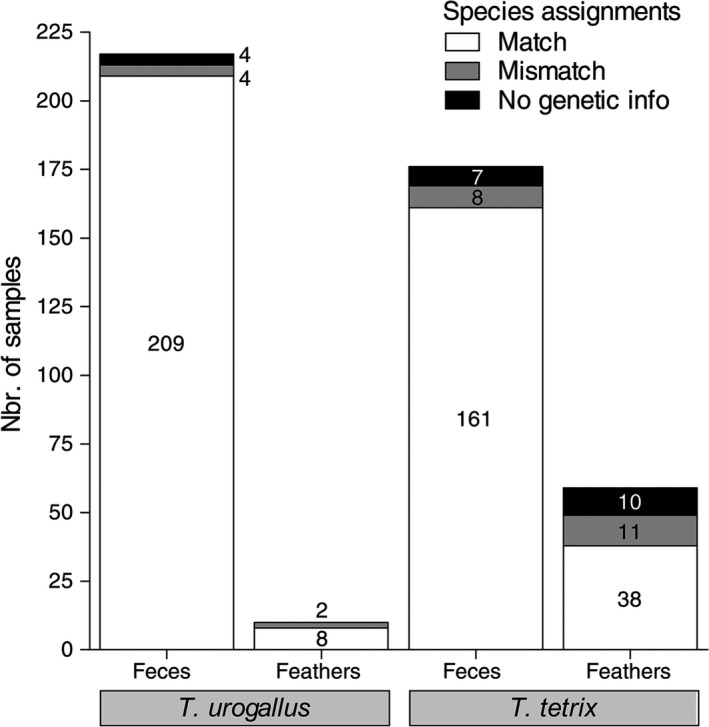
Genetic versus morphological species assignments of feces and feathers

The rate of correct morphology‐based species assignments was mainly influenced by the species of origin and sample type. Utilizing the molecular genetic species assignments as a reference, for capercaillie, 209 (96.3%) feces and eight (80.0%) feather samples were correctly attributed to their source species when using the morphological approach. For the black grouse specimens, these figures read 161 (91.5%, feces) and 38 (64.4%, feathers; Figure 3). Eight feces and five feather samples of capercaillie and black grouse were confused with each other. Moreover, the rate of proper assignments within the pool of black grouse samples was slightly reduced due to eight rock ptarmigan feathers and three feces samples originally believed to stem from black grouse. Both species carry similar, white feathers in their plumage and occur in overlapping habitats. Additionally, one feces sample believed to derive from black grouse was molecular genetically identified as hazel grouse. The nine samples with inconclusive morphological species assignment were not considered in this comparison.

#### 3.2.3 Reliability of genotyping

To test the reliability of analysis, PCR genotyping was repeated for twelve capercaillie and twelve black grouse samples six times each. In the initial round of STR genotyping, per species six of these samples yielded full (i.e., 8‐locus) profiles, while the other six specimens produced informative 5–7 locus genotypes only. For capercaillie, this test yielded a mean percentage for positive amplification over all loci of 98.8% for full and 86.4% for the partial profiles. For black grouse, the respective figures read 99.0% and 79.9%. In capercaillie, the rates of allelic dropout (ADO) and false alleles (FA) were 15.3% and 1.2% across all loci. For black grouse, these values were 16.8% (ADO) and 3.8% (FA, Table 3).

**Table 3:** Genetic parameters and rates of allelic dropout and false alleles for capercaillie and black grouse

	A	*SD*	G	*SD*	Pi	Pi_sib_	ADO	FA
Capercaillie	6.88	±2.70	0.67	±0.36	8.83 × 10^−8^	1.34 × 10^−3^	15.3%	1.2%
Black grouse	7.38	±2.07	0.58	±0.32	5.01 × 10^−8^	1.41 × 10^−3^	16.8%	3.8%

A, average number of alleles/locus; G, average gene diversity over loci; Pi, probability of identity; Pi_sib_, probability of identity for siblings; ADO, allelic dropout rate; FA, rate of false alleles.

#### 3.2.4 Individualization of samples

For individualization, only genotypes comprising at least five successfully amplified loci were used. This was the case in 205 (90.7%) capercaillie and 194 (92.4%) black grouse samples (Tables 4, S6 and S7). Establishing identity for profiles from multiple samples of an individual is not trivial when incomplete or otherwise flawed genotypes are present. Here, according to the routines implemented in the allelematch package, up to three pairwise mismatches due to allelic dropout were tolerated for both species. On the contrary, even a single mismatching heterozygous allelic state was considered probative for nonidentity. Additionally, samples with genotypes differing pairwise only in potentially missing alleles or featuring a single mismatching allele at a single heterozygous locus were re‐amplified and re‐analyzed (“targeted multiple tubes approach”). In the single instance of a capercaillie dropping, this procedure failed to unambiguously clarify an issue arising from supposed false alleles. To avoid the generation of a phantom individual, this particular profile was not considered unique.

**Table 4:** Samples analyzed and genotypes obtained for capercaillie and black grouse

	Capercaillie					Black grouse				
	*N*	*N*_8_ loci	%	*N*_5–8_ loci	%	*N*	*N*_8_ loci	%	*N*_5–8_ loci	%
Feces	215	183	85.12	197	91.63	170	125	73.53	156	91.76
Feathers	11	7	63.64	8	72.73	40	35	87.50	38	95.00
Total	226	190	84.07	205	90.71	210	160	76.19	194	92.38

*N*, number of samples molecular genetically identified as capercaillie or black grouse; *N*_8_ (*N*_5–8_) loci, number of samples where all eight (at least 5) loci showed detectable amplification; %, number of samples where all eight (at least 5) loci were amplified in percent.

One hundred and eighty‐three (85.1%) of the 215 feces and seven (63.6%) of the total 11 feather samples molecular genetically attributed to capercaillie yielded full STR profiles. For individualization, 205 (90.7%) of the 226 molecular genetically identified capercaillie samples were suitable, and 63 different genotypes were found and attributed to distinct individuals (Tables 4 and S6). Forty of these 63 STR profiles were found more than once, which resulted in an average of 3.25 ± 2.91 observations per individual. Pi and Pi_sib_ values for capercaillie genotypes were calculated as 8.83 × 10^−8^ and 1.34 × 10^−3^, respectively (Table 3).

For black grouse, 194 (92.4%) of the 210 specimens were considered informative by featuring at least five successfully amplified microsatellite loci (Table 4) and 125 (73.5%) of the 170 droppings and 35 of 40 feathers (87.5%) yielded 8‐locus STR profiles. Altogether, 114 different STR profiles were obtained, and for 33 birds, samples were repeatedly recovered. The average number of observations per individual was 1.70 ± 1.65. For black grouse, estimated Pi and Pi_sib_ values of 5.01 × 10^−8^ and 1.41 × 10^−3^ were obtained, respectively (Table 3).

The number of different alleles per locus ranged from 3 (sTuT2, black grouse) to 13 (sTuD6, capercaillie, Table 2). In capercaillie, the mean number of alleles per locus and the average gene diversity over loci were 6.88 ± 2.70 and 0.67 ± 0.36, respectively. For black grouse, these figures read 7.38 ± 2.07 and 0.58 ± 0.32 (Table 3). Allele frequencies for capercaillie and black grouse are provided in Tables S2 and S3. After Bonferroni correction for multiple hypothesis testing, statistically significant deviation from HWE was estimated in capercaillie for mTuT1. In black grouse, we detected a statistically significant departure from HWE at loci mTuT1, BG15, sTuD6, sTuD1, and sTuT4 (Table 2).

## 4 DISCUSSION

### 4.1 Species assignments

**Figure 4:**
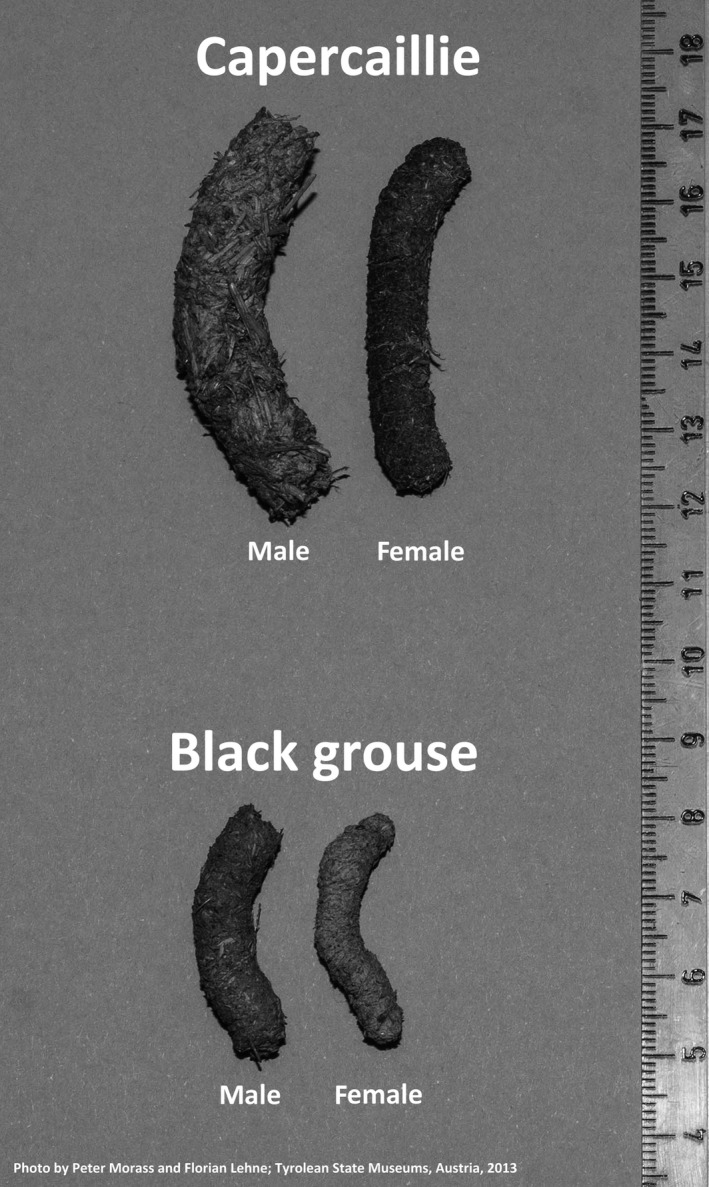
Feces of capercaillie (*Tetrao urogallus*) and black grouse (*Tetrao tetrix,* photograph Peter Morass and Florian Lehne Tyrolean State Museums, Austria)

When analyzing noninvasively collected feces and feather samples, reliable species‐of‐origin information is essential for data interpretation. For several grouse species, this information can be obtained by means of molecular genetic as well as morphology‐based approaches. At the molecular genetic level, species indicative allele size ranges at the STR loci BG15, BG18, and sTuT2 allow for allocation of feathers and feces samples to capercaillie, black grouse, hazel grouse, and rock ptarmigan (Jacob et al., 2010; G. Segelbacher, personal communication, February 12, 2013). This observation was confirmed by our data (Table 2). Likewise, differences in size and appearance of feces and feathers can facilitate ascertainment of species of origin and sex (Klaus, 1986, 1990). Feces of male capercaillie are usually thicker (10–12 mm) than those of female individuals of the same species (8–9 mm, Figure 4; Klaus, 1986). The latter, however, may be confused with droppings from male black grouse, which were reported at an average thickness of 9.5 mm (Klaus, 1990). Finally, female source droppings of black grouse are usually smaller with an average diameter of 7.7 mm (Klaus, 1990).

A comparison between the genetic and morphological approach for species assignment of feces and feathers revealed a high degree of consistency. Field collection was performed by experienced field surveyors, and especially for feces, the rate of correct assignments was very high. A few difficulties associated with feathers were observed for several reasons. Feathers of female capercaillie and black grouse can be mixed up due to similar coloration and pattern. More critically, however, the plumage of both male black grouse and rock ptarmigan contains small white feathers that can be confused with each other. This may cause problems, as the habitats of both species overlap (Klaus, 1990; Storch, 2007).

These aggravating factors have to be taken into consideration during the decision‐making process. The success rates of the morphology‐based approach, therefore, primarily depend on the experience level of field surveyors. However, if well‐trained personnel is unavailable, sample examination and specimen‐to‐species assignments may also be realized, for example, by an expert in the laboratory. In summary, it can be said that the morphological approach not only acts as gatekeeper for molecular genetic analyses but also makes for an important stand‐alone tool to establish the presence of particular species in a given habitat, particularly if financial resources and/or time for molecular genetic analyses are limited.

### 4.2 DNA sampling and extraction

We set out to optimize and streamline DNA extraction from feces with particular regard to nucleic acid yield and quality as well as diminished consumption of biological material, time, and labor. The feces surface layer was assumed to contain the highest concentration of epidermal cells and less PCR inhibitors. Hence, a dry swab from the surface could provide sufficient amounts of valuable DNA and simultaneously offer two significant advantages over the original procedure involving lysis of the specimens. Firstly, swabbing takes considerably less hands‐on time than processing entire feces samples or large portions thereof. This is of practical relevance when considering the high sample numbers usually required in ecological and conservation biological field studies. Secondly, only minute amounts of precious sample material are required by swabbing. Specimens were not destroyed for DNA extraction, and the larger portion remained available for potential use in further analyses or studies.

For feathers, we employed a DNA extraction approach aiming at challenging forensic hair samples, which includes a dedicated extraction buffer containing calcium (Hellmann et al., 2001). Use of this particular buffer, which was specifically designed for DNA extractions of keratinized cells, resulted in complete lysis of the feather samples. DNA from feathers mainly derives from remaining keratinized pulp caps and from adherent cells (i.e., epidermal cells) from the surface of the calamus (Horváth, Martínez‐Cruz, Negro, Kalmár, & Godoy, 2005). However, feathers, which have been exposed to adverse environmental conditions, may lack these adherent cells. Additionally, the DNA may already have been degraded, and DNA in keratin cells of the calamus may be the only source of DNA (Hellmann et al., 2001). Therefore, by digesting the whole feather tip, an additional source of DNA was made accessible, which may explain the observed increase in genotyping success.

Application of these improved protocols for DNA extraction is not restricted to the bird species analyzed here, but can be easily adapted for use on noninvasive samples from a broad range of animals.

### 4.3 Genotyping and suitability of STR markers

It is generally known in forensic genetics that minute amounts of DNA in tissues exposed to environmental stress show degradation with fragment sizes above 250 bp being significantly less abundant in the extract [e.g., (Grubwieser et al., 2006)]. Therefore, shortening amplicon sizes by moving primers closer to the repeat region (Jacob et al., 2010) is expected to increase the amplification success rate (Grubwieser et al., 2006). Accordingly, in our study, a high rate of 8‐locus profiles was achieved by testing polymorphic STR markers with multiplex PCR assays that yielded short amplicons (<220 bp). For capercaillie (*n* = 226) and black grouse (*n* = 210), 84.1% and 76.2% of the profiles showed detectable amplification at all eight loci, respectively (Table 4).

Furthermore, the optimized feather and feces extraction methods bolstered genotyping success. For both capercaillie and black grouse, more than 90% of the samples were suitable for genetic individualization based on the criteria applying in this study (Table 4). The success rate for capercaillie feathers was slightly reduced, maybe due to a stochastic effect arising from the low sample number. Eight of eleven samples (72.7%) qualified for individualization and seven (63.6%) yielded full STR profiles (Table 4).

The estimated Pi and Pi_sib_ values (Table 3) were within the threshold recommended by Waits et al. (2001), who defined values between 1 × 10^−2^ and 1 × 10^−4^ as reasonably low for ecological studies. Therefore, the applied system of eight STR loci appeared powerful enough to discriminate among individuals, even in the case of close relatedness (e.g., parent–child or sibling relationship).

In capercaillie and black grouse, a statistically significant deviation from HWE was observed at locus mTuT1. Jacob et al. (2010) detected the same issue in similar studies on capercaillie. In black grouse, four additional loci (BG15, sTuD6, sTuD1, and sTuT4) deviated statistically significantly from HWE. Generally, such a deviation can indicate inbreeding or a distinct population structure. Other possible explanations are poor DNA quality resulting in allelic dropout or primer‐binding site mutations causing reproducible null alleles. Yet, the short amplicon sizes generated with our approach and the observation that only particular loci were affected, rendered random dropout a rather unlikely explanation (Jacob et al., 2010). The software microchecker revealed the potential presence of null alleles for all loci deviating from HWE. In addition, the possibility of scoring errors caused by stutter bands of dinucleotide repeat markers cannot be completely excluded for sTuD6 and sTuD1 according to microchecker (data not shown). This class of microsatellites is known for its propensity to stutter peak formation, which can cause ambiguous allele calls (Butler, 2005; Taberlet et al., 1999). Nevertheless, due to their high capacity to discriminate among individuals (Table 2), all markers deviating from HWE were considered further on.

Based on GIMLET results, allelic dropout (ADO) and false alleles (FA) were the primarily observed error types (Table 3), as determined in the reliability testing experiment. In comparison with similar studies on capercaillie, the estimated error rates due to ADO (15.3%, Table 3), averaged over loci, were below the values reported by Regnaut, Lucas, and Fumagalli (2006) (21% ADO), but above those published by Pérez, Vázquez, Quirós, and Domínguez (2011) (8.1% ADO), whereas false allele error rates (1.2%, Table 3), again averaged over loci, were below values reported by both Regnaut et al. (2006) (3% FA) and Pérez et al. (2011) (1.4% FA). Unfortunately, pertinent data on black grouse were not available in the literature. Considering that the pool of samples used to estimate ADO and FA featured a 50% proportion of rather challenging profiles comprising 5–7 loci only, leads to the assumption that overall error rates were overestimated, as only 6.6% of the capercaillie and 16.2% of the black grouse samples in the main study showed such incomplete, but informative, profiles.

Due to low quantities of template DNA and/or DNA degradation, ADO is one of the most frequently reported error types when working with noninvasive sampling material. FA are mostly attributable to artefacts, although they can also indicate contamination (Taberlet et al., 1996). In the reliability test, the percentage of FA was very low. Therefore, it can be assumed that both artefacts and contamination were rare in the main study as well. This again highlights the suitability of our DNA extraction and multiplex PCR amplification approaches for analysis of feces and feather samples from capercaillie and black grouse.

### 4.4 Individualization of specimens

While being highly advantageous, noninvasive sampling of populations also entails analytical challenges. Due to the lack of a known one‐to‐one link between a specimen and an individual, multiple samples originating from a single animal may be analyzed. Thus, during data analysis, matching multilocus genotypes are pooled and collapsed into a unique consensus profile, which is subsequently attributed to a single, yet unknown, individual. In such an approach, genotyping error has to be accounted for to avoid creation of phantom individuals by declaring flawed STR profiles unique. To date, confirmation of genotyping results by a multiple tubes approach (Taberlet et al., 1996) represents the state‐of‐the‐art attempt in controlling genotyping error and obtaining the accurate number of unique genotypes in a data set. In a multiple tubes approach, STR profiles are challenged in several independent rounds of genotyping. Errors are then revealed by building a consensus genotype for each sample. In some cases, however, missing information for some loci will persist, and due to limited financial resources, large‐scale repeat PCR genotyping may not always be feasible. Moreover, global application of a multiple tubes approach appears not always to be absolutely necessary. On data sets containing low proportions of partial multilocus profiles and a considerable number of repeatedly sampled individuals, an alternative approach may be considered (Galpern et al., 2012). Under such circumstances, erroneous individualization due to ADO can be minimized by utilizing dedicated software, such as the allelematch package for R (Galpern et al., 2012). Here, clusters of identical or similar genotypes potentially arising from repeatedly sampling single individuals are identified on basis of a pairwise dissimilarity matrix (Tables S6 and S7, Galpern et al., 2012). Such clusters help to control for genotyping error, as they may be considered a mimic of genotype alignments obtained by a multiple tubes approach. In our study, random allelic dropout has been identified as the major source of error. For different samples originating from the same individual, pairwise comparison of genotypes may yield mismatching allelic states at loci being actually heterozygous (e.g., pseudo‐homozygous vs. actually heterozygous). Allowing for such dropout‐related pairwise mismatches, thus, will counteract this issue and help to reduce the number of phantom individuals. Nevertheless, to prevent assignment of a partial profile to multiple individuals, a maximum number of allowed pairwise mismatches have to be determined. This optimization process is aided by the allelematch software (Galpern et al., 2012). As the output from the allelematch software helps in pinpointing potentially erroneous STR profiles, it may be used in a “targeted multiple tubes” approach focusing on these questionable genotypes only.

False alleles were found to be much rarer than random allelic dropout events. Nevertheless, they constitute another form of genotyping error that may inflate the number of phantom individuals. To counteract this, all multilocus genotypes pairwise differing by a single allele at a single locus only were challenged by re‐amplification and re‐analysis of the affected samples.

For data sets with a low expected proportion of multiple sampling and/or a high overall error rate, the “global” multiple tubes approach undoubtedly will be advantageous. Ultimately, depending on the design and aim of the study, it has to be evaluated, whether a global multiple tubes approach is required or if a software‐based solution tied to target retyping of questionable samples appears sufficient.

## 5 CONCLUSIONS

This study describes an optimized DNA extraction method for the molecular genetic analysis of noninvasive samples including feces and feathers. Importantly, the risk of sample cross‐contamination and mix‐up was minimized and the DNA extracts proved suitable for successful downstream genotyping applications. Multiplex PCR assays producing small amplicons from informative STR markers were developed. Additionally, advanced software solutions were used for analyzing genotyping data featuring missing information at some loci and a mild amount of false alleles. This approach fostered reliable genotyping of capercaillie and black grouse individuals. In addition to this, we are confident that our protocols will foster the use of noninvasively collected specimens in ecological and conservation studies, as they can be easily applied to a broad range of animal species.

## CONFLICT OF INTEREST

None declared.

## AUTHOR CONTRIBUTIONS

SV, HN, BB, RL, and WP participated in study design. SV and RL collected the samples. SV and HN extracted DNA. SV, HN, BB, RL, and WP analyzed the data. SV, HN, and WP drafted the manuscript. All authors revised and added to the draft. All authors reviewed and approved the final manuscript.
