# Supplementary material for: Increased DNA typing success for feces and feathers of capercaillie (Tetrao urogallus) and black grouse (Tetrao tetrix)
Source: Ecol Evol. 2018 Mar 23;8(8):3941–51. doi: 10.1002/ece3.3951 (PMC5916295; doi:10.1002/ece3.3951)
Supplement: Supplementary file 10 [file ECE3-8-3941-s010.docx]

# S Appendix 1

# Code for the “allelematch” script for R

Sys.time**()**

rm**(**list**=**ls**())** ## "reset" R

## setwd("workingdirectory") ## set R's current working directory as desired

setwd**(**"workingdirectory"**)** ## set R's current working directory as desired

getwd**()** ## verify R's current working directory

amGROUSE **<-** read.csv**(**"amGROUSE.csv", sep**=**";", header**=TRUE)**

## ls() ## list all objects

## verify correct data was imported (e.g. check for extra cols or rows)

## names(amGROUSE) ## returns column names

## str(amGROUSE) ## a compilation of the above

## fix(amGROUSE)

library**(**allelematch**)**

## amGROUSE <- amDataset(amGROUSE, indexColumn="sampleID", missingCode="-99")

amGROUSE **<-** amDataset**(**amGROUSE, indexColumn**=**"sampleID", missingCode**=**"-99"**)**

amUniqueProfile**(**amGROUSE, doPlot**=TRUE)**

uniqueGROUSE **<-** amUnique**(**amGROUSE, alleleMismatch**=**3**)**

summary**(**uniqueGROUSE, html**=**"uniqueGROUSE.html"**)**
